# Opportunity for cost savings with a novel differentiated model of PrEP delivery: a comparative costing analysis of six-month PrEP supported by interim HIV self-testing and standard of care PrEP dispensing in Kenya

**DOI:** 10.1186/s12913-025-12891-7

**Published:** 2025-07-01

**Authors:** Dorothy I. Mangale, Jesse Heitner, Katrina F. Ortblad, Peter Mogere, Catherine Kiptinness, Nelly R. Mugo, Jared M. Baeten, Kenneth Ngure, Ruanne Barnabas

**Affiliations:** 1 Department of Global Health, University of Washington, Seattle, USA; 2 Mass General Brigham, Boston, USA; 3 Public Health Sciences Division, Fred Hutchinson Cancer Center, Seattle, USA; 4 Center for Clinical Research, Kenya Medical Research Institute, Nairobi, Kenya; 5 Department of Medicine, University of Washington, Seattle, USA; 6 School of Public Health, Jomo Kenyatta University of Agriculture and Technology Nairobi, Nairobi, Kenya; 7 Division of Infectious Diseases, Massachusetts General Hospital, Boston, USA; 8 International Clinical Research Center, University of Washington, 908 Jefferson St, 12th Floor, Seattle, WA 98104 USA

**Keywords:** Pre-exposure prophylaxis (PrEP), Differentiated service delivery (DSD), Cost analysis, Multi-month dispensing, HIV self-testing (HIVST), Sub-Saharan Africa (SSA)

## Abstract

**Background:**

Cost remains an important barrier to HIV pre-exposure prophlyaxis (PrEP) delivery in Africa. Simplified delivery models that reduce costs without compromising PrEP outcomes are needed. The JiPime-JiPrEP trial tested a model of six-month PrEP dispensing supported with interim HIV self-testing (HIVST) and found non-inferior HIV testing, PrEP refilling, and adherence compared to three-month PrEP dispensing and quarterly clinic visits, the standard-of-care (SOC). We estimated the cost of this novel differentiated PrEP delivery model compared to SOC in Kenya.

**Methods:**

Using activity-based micro-costing (payer perspective) and time-and-motion observations, we estimated the cost of PrEP delivery (per client-month) in the intervention and SOC between May 2018 to December 2019. Data from budgets and expense reports, published documents, and interviews informed our estimates. We calculated costs over a one-year horizon for: 1) the *trial scenario* (i.e., costs within the trial), and 2) the *Ministy of Health (MOH) scenario* (i.e., hypothetical costs at public clinics). Estimates were in 2019 US dollars and excluded research-related costs.

**Results:**

The mean personnel time attributable to PrEP delivery was 76 minutes per visit and 152 minutes projected over a year in the intervention and 54 minutes per visit and 282 minutes per year in the SOC. In the trial scenario, PrEP delivery cost $17.73 per client-month in the intervention (n=2039 PrEP-months) and $25.50 in the SOC (n=913 PrEP-months). The projected cost of PrEP delivery in the MOH scenario was $11.94 in the intervention and $14.76 in the SOC, with the addition of HIVST kits in the intervention more than offset by personnel savings. In this scenario, personnel (intervention: 55%; SOC: 44%) and medication (intervention: 16%; SOC: 32%) were the primary cost drivers. Including serum creatine testing twice a year in the MOH scenario resulted in a slight increase in the cost of PrEP delivery in the intervention ($12.88 per client-month) versus SOC ($16.17 per client-month).

**Conclusions:**

Six-month PrEP with interim HIVST demonstrated lower costs than three-month dispensing, with decreased personnel time. Scale-up of PrEP delivery requires efficient use of limited resources; the savings in this model of PrEP delivery could be redirected towards currently unmet medical needs.

**Clinial trial number:**

NCT03593629||https://www.clinicaltrials.gov/ with the Clinical Trial Registry (Registration date: 2018-07-20).

**Supplementary Information:**

The online version contains supplementary material available at 10.1186/s12913-025-12891-7.

## Introduction

Differentiated models of daily oral HIV pre-exposure prophlyaxis (PrEP) delivery are currently being developed and tested to support PrEP scale up throughout sub-Saharan Africa [1, 2]. A differentiated service delivery (DSD) model is a strategy designed to meet the specific needs of a defined target group of patients or clients by accounting for specific situations, preferences, and clinical needs of the group [3–6]. Differentiated models are intended to mitigate PrEP delivery challenges primarily related to client preferences i.e. barriers related to when, where, who, what and how often to collect PrEP from facilities, but also human resource needs, financing and commodity shortages, and organizational characteristics that influence PrEP uptake and the sustainment of PrEP programs [7–11]. Understanding the costs of these new delivery models (e.g., pharmacy-based PrEP delivery [12, 13], multi-month PrEP dispensing [14]) is critical to determining their sustainability in real-world settings. Currently in most sub-Saharan African settings, including Kenya, PrEP is primarily delivered at clinics within public healthcare facilities, including HIV [15–18], maternal and child health, and family planning clinics [19–21]. Cost is an important barrier to PrEP access and delivery in these settings [22, 23]. PrEP clients incur costs traveling to and waiting at (i.e., spending time away from work) the clinics, while health systems incur costs, such as personnel time (i.e., for counseling PrEP clients) and medical supplies (e.g., HIV testing kits, PrEP drugs) at the clinics.

Previous studies evaluating the cost of PrEP delivery at public clinics in Kenya have found that the projected cost of implementation, although affordable, could be improved upon to make room for newer, more efficient delivery approaches [24–27]. For example, the economic cost of MOH-delivered PrEP for all people at HIV risk in public HIV clinics was estimated to be $10.31 US dollars (USD) per person-month ($123.72 USD per person-year) [26] and the additional cost of adding PrEP delivery for HIV serodifferent couples receiving care in this setting was $7.23 USD per couple per month ($86.79 USD per couple per year) [24]. Additionally, the cost of integrating PrEP into routine public maternal and child and family planning services was averaged $15.00 USD per client-month ($180 USD per person-year) [27, 28]. With Kenya government’s average health expenditure of $83 USD per capita [29], intended to include coverage for services beyond HIV, more efficient models of PrEP delivery that minimize costs to the health system while maximizing exisiting resources and infrastructure, are needed.

One innovative model of PrEP delivery that may reduce client and health system costs is six-month PrEP dispensing with interim HIV self-testing (HIVST) at three months to reduce the number of annual PrEP clinic visits in half. A recent randomized implementation trial found that this intervention did not compromise recent HIV testing, PrEP refilling, or PrEP adherence outcomes compared to standard PrEP delivery with three-month dispensing [30]. In this economic evaluation, we aim to measure the cost of this new PrEP delivery model both in the context of the research study and hypothetical context of real-world public clinics. Findings from this analysis will help establish the affordability and sustainability of six-month PrEP dispensing supported with HIVST and inform the design of other streamlined PrEP delivery approaches.

## Methods

### Summary of the JiPime-JiPrEP trial

The JiPime-JiPrEP trial was a three-group non-inferiority randomized trial conducted at the Partners in Health and Research Development (PHRD) Research Clinic in Kiambu County, Kenya (ClinicalTrials.gov: NCT03593629, 2018-07-09) [17]. Participants were recruited from Kiambu Country and the surrounding counties where population-level HIV prevalence is ~4% [31]. Individuals were eligible for enrollment if they were ≥18 years, tested HIV-negative on a rapid test, had been using PrEP for at least one month and planned on continuing PrEP use, and were willing to provide written informed consent [14, 32].

Participants were randomized 2:1 to: 1) *six-month PrEP dispensing* supported by HIVST at three months (with semi-annual clinic visits), or 2) *standard-of-care (SOC) PrEP delivery* (three-month PrEP dispensing with quarterly clinic visits). Those in the intervention group were additionally randomized 1:1 to receive either oral-fluid (OraQuick Rapid HIV-1/2 Antibody Test, OraSure Technologies, Bethlehem, USA) or blood-based (Mylan Rapid HIV 1&2, Mylan Laboratories. Ltd., Canonsburg, USA) HIVST kits. At enrollment, participants randomized to the intervention group were dispensed six months of PrEP, two HIV self-tests, and instructed to return to the clinic every six months for follow up. Participants in the SOC group were dispensed three months of PrEP, received no HIV self-tests, and were instructed to return to the clinic every three months. All PrEP clinic visits were conducted according to Kenya’s PrEP delivery guidelines, which included clinical examination, HIV risk and PrEP adherence counseling, HIV testing (using blood-based rapid tests), serum creatinine clearance testing, syndromic management of sexually transmitted infections, and urine pregnancy testing [33]. All participants were followed for 12 months. Research ethics review committees at the University of Washington and Kenya Medical Research Institute examined and approved the protocol. More details on this study are published elsewhere [14, 32].

### Costing approach

We performed activity-based micro-costing [28] following published guidelines [34, 35] to estimate the average cost per client-month of PrEP delivered. We calculated the total annual cost of PrEP delivery divided by the months of PrEP dispensed in a year by study group. We grouped costs into fixed and variable costs, and by type of cost input (see Supplementary material). Fixed costs included the value of resources associated with start-up training, demand creation, supervision and administration, capital, and overheads. Variable costs were those that changed with the volume of client services, such as personnel time for clinical assessments, PrEP drugs, clinical and laboratory supplies, and refresher trainings.

### Data collection

We used data from time-and-motion observations, trial accrual reports, interviews with key informants from the Kenya Ministry of Health (e.g. procurement officers, county health officials) and PHRD staff, and government price lists [36, 37]. Personnel, utility, supplies, equipment, and other capital and overhead costs from the PHRD clinic were extracted from study expense reports, budgets, and interview responses (see guide included in supplemental material). Variable costs were grouped into three main activity groups; costs associated with: 1) the delivery of routine clinical care (including counselling services, and physical examination), 2) testing services (including HIV testing and HIVST kits), and 3) PrEP dispensing (including PrEP drugs). Costs related to research activities were excluded from the analysis.

Time-and-motion observations were completed between July to September 2019 at the PHRD research clinic. We observed participants throughout their PrEP clinic visit and recorded the start and end times of different visit activities; periods of inactivity were recorded as waiting time. To obtain a representative sample, we varied the times of day observations were recorded and followed different staff members. To estimate PrEP visit accruals and drugs dispensed, we used data from implementation of the JiPime-JiPrEP trial (collected from May 2018 to December 2019), where participants in the intervention group received two annual visits and six PrEP bottles per visit, and those in the SOC group received four annual visits and three PrEP bottles per visit. To estimate the unit cost for PrEP, oral-fluid HIVST kits, and blood-based HIVST kits (Table 1), we used information from published studies and interviews; costs associated with procurement (e.g., warehousing and distribution) were included. All costing data was collected in 2019 USD or Kenyan shillings (KES), which we converted to USD at an exchange rate of 1 USD per 105.50 KES [38].

**Table 1:** Key input assumptions for estimation of the cost of PrEP delivery

Personnel	
* Trial scenario*	
Salaries (per annum, inflation adjusted)	
HIV Testing Counsellor	$9,365
Clinical Officer	$10,841
Nurse	$9,984
Pharmacy technologist	$10,085
* MOH scenario*	
Salaries (per annum, inflation adjusted)	
HIV Testing Counsellor	$3,226
Clinical Officer	$7,290
Nurse	$6,101
Pharmacy technologist	$6,001
Weekdays worked per annum	231
Medication, lab & supplies	
PrEP (30 days TDF/FTC)	$6.75
Oraquick HIV ST	$4.27
Atomo HIV ST	$6.17
Determine HIV Rapid Test	$0.83
Creatinine test- MOH scenario	$2.51
*Exchange rate (2019 $US)*	*$1 USD = 105.35 KES*
*Inflation rate*	*4.40%*
*Discount rate*	*3.00%*

### Unit and total cost estimates

To estimate the total cost of PrEP service delivery, we summed annualized fixed and variable costs incurred in one year of PrEP provision using the number of PrEP-months dispensed per client-month (1 PrEP refill per month = 1 bottle of 30 pills) as a denominator for calculating unit costs.

To estimate variable unit costs of personnel time accrued by providers, we divided providers’ annual salaries by the total number of hours worked annually (2,080 hours) to establish an hourly wage. We calculated the variable cost of PrEP activities by applying personnel salaries to the average time spent per activity, for each cadre. To estimate laboratory costs, we multiplied the associated unit cost of tests and consumables used with the number of clinic visits accrued, by study group. We included rapid HIV tests and HIVST kits in the laboratory costs. The total cost of drugs dispensed was estimated using the unit price of PrEP and the number of PrEP months accrued within the one-year period. To calculate the fixed costs for each arm, we multiplied the total cost of fixed inputs by the fraction of total PrEP visits accrued in each arm.

### Analysis

We estimated PrEP delivery costs gathered from May 2018 to December 2019 to reflect resource use in two scenarios: 1) the *trial scenario,* that reflects the actual implementation cost of the JiPime-JiPrEP trial, and 2) the *MOH scenario,* which reflect hypothetical costs if the MOH were to implement six-month PrEP dispensing with HIVST at public clinics. We annualized all capital, start-up, and training costs assuming 1–5 years of useful life (5 years for equipment and other capital goods, 1 year for training) and applied a 3% annual discount rate.

#### Trial scenario

In the trial scenario, we estimated costs in the controlled context of a trial conducted at a well-established research site that operates with considerable efficiency owing to extensive experience with PrEP delivery. We estimated the cost of PrEP delivery for all participants who completed a clinic visit and were dispensed PrEP. Personnel costs (calculated from annual salaries) included the time research staff spent delivering PrEP clinical services and excluded the time they spent on research-related activies (see Supplementary material). PrEP delivery costs included the cost for counselling, clinical examination and treatment by clinicians, HIV testing and PrEP dispensing. We excluded costs associated with testing for pregnancy, STIs, or any other test indicated by the clinician during examination, as these are not included national PrEP delivery guidelines [33, 39]), and PrEP dispensing.

#### MOH scenario

In the MOH scenario, we projected costs of the intervention if delivered in public HIV clinics operated by the Kenyan MOH. To estimate the personnel costs associated with the delivery of PrEP clinical services, we replaced PHRD research staff salaries with public sector salaries and allowances. For personnel costs attributed to start-up, we included costs associated engaging with clinic leaders to approve the intervention, and training and supervising staff engaged in the delivery of PrEP services. We assumed each PrEP clinic visit (n=5 in total) lasted approximately two hours. We included in start-up costs and the cost of microplanning meetings by estimating personnel time of county health officials, cost of travel, and other per diem costs spent travelling to facilities to introduce the intervention. We applied the same cost of PrEP delivery (including drug supply and HIV tests) as that used in the trial scenario. We obtained information on quantitites of supplies such as stationery and clinic consumables used for PrEP delivery from internal PHRD expense reports and interviews (see Supplementary material for guide).

The national PrEP delivery guidelines include a recommendation for serum creatinine testing for PrEP users, however, to remove barriers to PrEP initiation and continuation, absence of this test does not prevent PrEP delivery. Nonetheless, it is implemented at some facilities. Thus, as a sensitivity analysis, we applied the same estimation approach as the MOH scenario above and included the costs associated with serum creatinine testing. We incorporated the cost of laboratory equipment, supplies and personnel costs associated with the creatinine test being conducted twice annually at public facilities to estimate the unit costs of PrEP delivery for each study arm.

## Results

Between May 2018 to December 2019, the JiPime-JiPrEP trial accrued 644 total PrEP clinic visits: 304 enrollment and 341 follow-up visits (87 three-month, 209 six-month, and 45 nine-month visits). There were 2,952 months of PrEP coverage (SOC: 913; intervention: 2,039) during the period selected for costing analysis (~60% of trial numbers). PrEP dispensing occurred at >99% of the observed visits (638/644).

We completed 22 time-and-motion observations (SOC: 9; intervention:13) between June and September 2019; six at PrEP enrollment visits and 16 at PrEP follow-up visits (Table 2). The average time it took providers to complete activities related to PrEP service delivery differed by study group. The mean time providers spent delivering services was longer per visit in the intervention (76 minutes, standard deviation [SD] 14 minutes) compared to the SOC (54 minutes, SD 10 minutes), but shorter when projected over the course of the year in the intervention (152 minutes, SD 29 minutes) compared to the SOC (282 minutes, SD 41 minutes).

**Table 2:** Distribution of personnel time spent by personnel responsible for PrEP delivery

	Both study arms	
	Median time per visit (IQR)	
Enrollment visit	*N*=6*	
Counselling & HIV Testing	33 (30 - 36)	
Clinical Examination	20 (9 - 25)	
Laboratory testing	5 (3 - 7)	
PrEP dispensing	21 (12 - 35)	
Total	79 (54 - 103)	

	SOC	Six-month PrEP + HIVST
	Median time per visit (IQR)	Median time per visit (IQR)
Refill visit (3, 6 & 9 month)	*N*=8	*N*=8
Counselling & HIV Testing	25 (24 - 36)	32 (25 - 35)
Clinical Examination	17 (10 - 25)	16 (10 - 22)
Laboratory testing	3 (3 - 5)	5 (3 - 9)
PrEP dispensing	6 (5 - 12)	20 (9 - 30)
Total	51 (42 - 68)	73 (43 - 96)

	Mean duration per visit (SD)	Mean duration per visit (SD)
All visit types	*N*=9	*N*=13
Counselling & HIV Testing	27 (3)	32 (3)
Clinical Examination	17 (4)	18 (4)
Laboratory testing	3 (1)	5 (2)
PrEP dispensing	7 (2)	21 (6)
Total	54 (10)	76 (14)

	Mean visit time per year (SD)	Mean visit time per year (SD)
All visit types	*N*=9	*N*=13
Counselling & HIV Testing	107 (13)	65 (6)
Clinical Examination	71 (16)	36 (8)
Laboratory testing	18 (5)	10 (3)
PrEP dispensing	26 (7)	41 (12)
Total	222 (41)	152 (29)

Values presented are rounded up or down to the largest whole number

Per year projections computed by multiplying SOC per visit times by 4 and intervention per visit by 2

Laboratory time excludes lab technician personnel time

*Only one enrollment visit observed In the SOC and five in the intervention, therefore we merged observations since enrollment procedures were the same

**Fig. 1:**
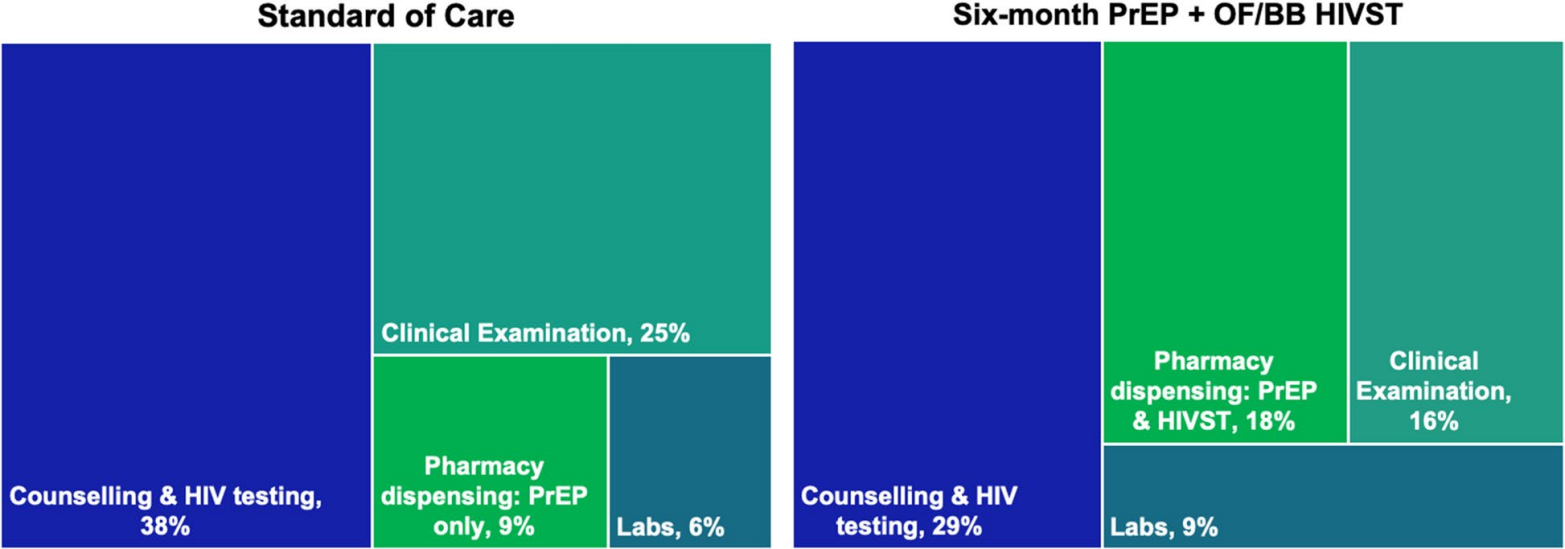
Distribution of average personnel time spent on PrEP delivery activities

Looking at activities constituting a single PrEP visit, the highest burden on personnel time was counselling (38%) and clinical examination (25%) in the SOC, and counselling (29%), pharmacy dispensing of PrEP and HIVST (18%), and clinical examination (16%) in the intervention (Fig. 1). The relative proportion of time for personnel spent on pharmacy dispensing was twice as high in the intervention (18%) compared to the SOC (9%). This was likely attributable to pharmacists having to review the HIVST instructions and demonstrate of how to use the kits, in addition to providing information on how to take PrEP effectively.

### Cost of PrEP provision: the trial scenario

**Fig. 2:**
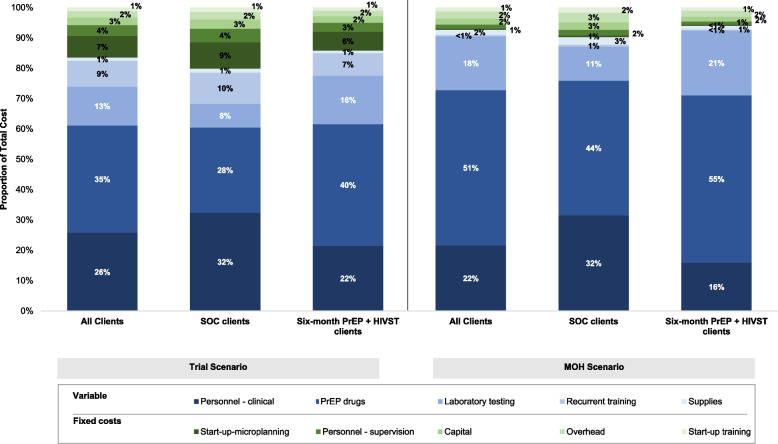
Distribution of total PrEP delivery costs by cost category, by study group and the different costing scenarios

The total economic cost of implementing six-month PrEP dispensing supported with interim HIVST in the trial scenario was $36,144 USD in the SOC compared to $23,284 USD in the intervention (Table 3). The primary drivers for economic costs in this scenario were clinical personnel (32%) and PrEP drugs for the SOC (28%), and PrEP drugs (40%), clinical personnel (22%), and laboratory testing (16%) for the intervention (Fig. 2). PrEP drugs and personnel-related costs were attributable for most variable costs in both groups (SOC: 70%; intervention: 66%). Start-up microplanning costs contributed the highest proportion of the fixed costs in both groups (SOC: 43%; intervention: 44%).

**Table 3:** Annual total cost and unit cost of PrEP per client-month in (2019 USD) for the trial scenario (2019 USD)

	Standard of care PrEP client visits (*n*=304); PrEP months (*n*=913)			Six-month PrEP + HIVST PrEP client visits (n=340); PrEP months (*n*=2039)		
Cost Categories	Total annual cost	$ per client/month	% of total cost	Total annual cost	$ per client/month	% of total cost
Variable costs						
Personnel (clinical)	$7,539.81	$8.26	32%	$7,774.92	$3.81	22%
PrEP drugs	$6,547.27	$7.17	28%	$14,486.20	$7.11	40%
Laboratory testing	$1,812.91	$1.99	8%	$5,773.97	$2.83	16%
Recurrent training	$2,398.93	$2.63	10%	$2,677.64	$1.31	7%
Supplies	$3,15.98	$0.35	1%	$308.13	$0.15	1%
*Sub-total*	*$18,614.91*	*$20.38*	*80%*	*$31,020.86*	*$15.22*	*86%*
Fixed costs						
Start-up-microplanning	$2,013.09	$2.20	9%	$2,246.98	$1.10	6%
Personnel (supervision)	$1,035.62	$1.13	4%	$1,067.04	$0.52	3%
Capital	$707.62	$0.77	3%	$789.83	$0.39	2%
Overhead	$580.01	$0.64	2%	$647.40	$0.32	2%
Start-up training	$333.50	$0.37	1%	$372.24	$0.18	1%
*Sub-total*	*$4,669.83*	*$5.11*	*20%*	*$5,123.49*	*$2.51*	*14%*
Total costs	$23,284.74	$25.50	100%	$36,144.35	$17.73	100%

In the trial scenario, the estimated average cost of providing one month of PrEP per client-month was $25.50 USD in the SOC, and $17.73 USD in the intervention, with a ~25% or $8.30 USD difference between groups in favor of the intervention (Table 3). Notably, the unit cost per client-month associated only with clinical personnel time was lower in the intervention ($3.81 USD) compared to the SOC ($8.26 USD), while the unit cost associated only with pharmacy dispensing activities was lower in the SOC ($1.99 USD) versus the intervention ($2.83 USD).

### Cost of PrEP provision: MOH scenario

In the MOH context, the total economic cost of implementing six-month PrEP dispensing supported with interim HIVST was $14,763 USD in SOC compared to $26,263 USD in the intervention (Table 4). These costs were 27% less in the intervention and 37% less in the SOC compared to the respective costs estimated in the trial scenario. Clinical personnel (50%) and PrEP drugs (22%) were the primary cost drivers of total economic costs. The proportion of clinical personnel costs in the SOC (32%) was double that of the same costs in the intervention (16%). The relative contribution of laboratory costs to the economic cost of PrEP delivery in the intervention (21%) was approximately two times the contribution of the same in the SOC (11%). In both groups, variable costs made up most of the economic costs (SOC: 90%; intervention: 94%), with a small proportion of the total (<3%) attributable to fixed costs.

**Table 4:** Annual total cost and unit cost of PrEP per client-month for the MOH scenario (2019 USD)

	Standard of care PrEP client visits (N=304); PrEP-months (N=913)			Six-month PrEP + HIVST PrEP client visits (N=340); PrEP-months (N=2039)		
Cost Categories	Total annual cost	$ per client/month	% of total cost	Total annual cost	$ per client/month	% of total cost
Variable costs						
Personnel (clinical)	$4,657.48	$5.10	32%	$4,186.13	$2.05	16%
PrEP drugs	$6,547.27	$7.17	44%	$14,486.20	$7.11	55%
Laboratory testing	$1,656.97	$1.81	11%	$5,599.91	$2.75	21%
Recurrent training	$89.24	$0.10	1%	$99.60	$0.05	0.4%
Supplies	$376.32	$0.41	3%	$324.26	$0.16	1%
*Sub-total*	*$13,327.28*	*$14.59*	*90%*	*$24,696.11*	*$12.11*	*94%*
Fixed costs						
Start-up-microplanning	$79.31	$0.09	1%	$88.53	$0.04	0.3%
Personnel (supervision)	$277.32	$0.30	2%	$274.56	$0.13	1%
Capital	$375.06	$0.41	3%	$418.64	$0.21	2%
Overhead	$468.96	$0.51	3%	$523.45	$0.26	2%
Start-up training	$235.35	$0.26	2%	$262.70	$0.13	1%
* Sub-total*	*$1,436.01*	*$1.57*	*10%*	*$1,567.87*	*$0.77*	*6%*
Total costs	$14,763.29	$16.17	100%	$26,263.98	$12.88	100%

In the MOH scenario, the unit cost of PrEP delivery for a single client per month was $16.17 USD in the SOC, and $12.88 USD in the intervention (Table 4). The clinical personnel unit costs per month in the SOC ($5.10 USD) was more than double that in the intervention ($2.05 USD), while the laboratory unit costs per month in the intervention ($2.75 USD) were roughly double that in the SOC ($1.81 USD).

Looking at the relative distribution of costs, the cost attributable to PrEP drugs, clinical personnel, and laboratory testing were primary costs drivers in both scenarios and groups (Fig. 2). Overall, costs associated with PrEP drugs were attributable to largest proportion of total costs in both scenarios (research: 35%; MOH: 51%) and in both secnarios was higher in the intervention (research: 40%; MOH: 55%) compared to the SOC (research: 28%; MOH: 44%). Across the scenarios, the proportion of total cost attributable to clinical personnel was lower in the research (22%) versus MOH (16%) scenarios for the interveniton but did not change by scenario for the SOC (32% in both). Additionally, laboratory costs were important drivers of total PrEP delivery costs, especially in the MOH scenario, where these costs were 18% of total costs compared to 13% in the SOC. Across the board fixed costs were small; <20% of total costs in the trial scenario and <10% of total costs in the MOH scenario.

Integrating bi-annual serum creatinine testing during PrEP delivery resulted in slight increases in the average unit cost of PrEP delivery in the MOH scenario: $16.17 USD per client-month in the SOC versus $12.88 USD per client-month in the intervention (Table 5, Appendix).

**Table 5:** Summary of unit cost per client-month of PrEP

Cost per client-month of PrEP	Standard-of-care	Six-month PrEP + HIVST	Cost difference (in $)	Cost difference (%)
As implememented *(without creatinine testing)*	$25.50	$17.73	$7.77	30%
MOH scenario *(without creatinine testing)*	$14.76	$11.94	$2.82	19%
MOH scenario *(with creatinine testing)*	$16.17	$12.88	$3.29	20%

## Discussion

The present findings demonstrate that six-month PrEP dispensing supported with interim HIVST is more efficient and less costly than SOC three-month PrEP delivery, while producing equivalent client outcomes [32]. The expense incurred by distributing HIVSTs alongside PrEP was more than offset by the cost of additional personnel time incurred by quarterly clinic visits under the SOC. Costs savings of this intervention were consistent in both the research and MOH scenarios, with greater cost savings in the latter.

Although dispensing HIVST alongside PrEP in the intervention introduced new costs to PrEP delivery, these were offset by the cost saving associated with halving the number of annual PrEP clinics with at-home HIVST. In the MOH scenario, we found a cost difference of -$2.82 USD or −24% in total PrEP delivery costs per client-month with the intervention compared to the SOC. Cost savings in PrEP delivery can be leveraged to increase equity in HIV programs by expanding PrEP access, establishing mechanisms for program sustainability and scale-up, freeing up funds to drive innovation and to encourage more support from policy makers [2, 40, 41]. Personnel time was an important input in our analysis which, alongside the additional cost HIVST kits, drove most of the differences in unit costs estimates between the study groups in both scenarios. This observation held despite the average duration of a single SOC visit being shorter than an average intervention visit, due to less time spent with pharmacists. In the intervention, PrEP dispensing at the pharmacy also included an instructional video demonstrating how to use the HIVST kit and reviewing with clients the steps required for accurate HIVST. The extra cost of HIVST kits was offset by personnel savings incurred by the halving number of annual PrEP clinic visits in the intervention compared to the SOC. Counselling activities add a substantial amount of personnel time at each PrEP visit and while these are important for PrEP initiation, they may be less important for continuation visits among clients established in care famliar with counseling messages. However, with fewer follow-up visits there may be an increased risk of missing vital associated services, like pregnancy testing and screening for common sexually transmitted infections.

Costs established in this study may inform affordability comparisons across other types of PrEP delivery models. We estimated that unit costs of six-month PrEP dispensing with interim HIVST at public MOH HIV clinics were $11.94 USD per client-month without serum creatinine testing, and $12.88 USD per client-month when bi-annual serum creatinine testing was included. These estimates are lower than estimates of other PrEP delivery models available in Kenya [24, 28]. The comparative affordability of this novel PrEP delivery model indicates it could be a viable option in resource-limited settings or where there are constraints on HIV services. Furthermore, the cost data gathered in this study can be applied to economic evaluations including budget impact analyses to support planning and prioritization, identify potential targets for cost reduction [42, 43].

There were some limitations to this analysis. This study benefited from leveraging a site with pre-existing resources, and infrastructure to support PrEP delivery, including well-trained and readily available staff, a good reputation in the community and an active community advisory board. We expect that the level of expertise and resource availability in public clinics will be variable and may result in lower efficiency of service delivery. However, the results from this implementation trial shed light on what may be possible. Secondly, we did not observe the MOH scenario, so the actual costs may be higher or lower depending on the contextual realities of the clinics (with overburdened providers contributing to inefficiencies [2]). In this costing analysis, unit costs were calculated based on records of PrEP dispensing and not adherence. We also did not account for PrEP discontinuation which is common among clients whose risk of HIV aquisition may vary over time [44]. To date, few studies have examined how start-and-stop models of PrEP use may impact the cost of PrEP delivery but we can speculate that where new PrEP initiation visits are needed, the cost of PrEP delivery might be higher [45, 46]. Additionally, due to time and resource constraints, we were not able to conduct time and motion observations with a larger sample of clients. This may reduce the generalizability of our findings and may not fully reflect the maximum cost incurred during PrEP client visits. However, we believe that our results are still reliable given the comparability of the unit costs we established relative to similar work done in other PrEP studies in Kenya. Lastly, we did not estimate the costs PrEP delivery incurred by clients as this analysis was conducted from the payer perspective, limiting our ability to examine the multitude of client factors that may influence PrEP use and subsequently PrEP delivery costs.

## Conclusion

Six-month PrEP with interim HIVST testing resulted in lower personnel requirements and lower overall delivery costs. The cost of adding HIVST kits to support PrEP delivery was offset by halving the annual number of PrEP clinic visits. This intervention is an innovative and economical way to increase efficiencies and streamline models of PrEP delivery without jeapordizing clients’ PrEP clinical outcomes. These findings are timely as African countries with high HIV burden wrestle with expanding PrEP coverage on limited health budgets. Scale-up of PrEP delivery requires the best use of limited resources, and the cost savings and personnel time evaluated in this analysis could be redirected to finance other unmet needs. Furthermore, as the PrEP landscape evolves with the introduction of new PrEP forms (e.g., injectables, vaginal rings), having more efficient models of PrEP delivery will help maintain advantages of oral PrEP among these forms.

## Supplementary Information

Supplementary Material 1.

Supplementary Material 2.

## Data Availability

Cost data utilized in this analysis are available upon request by contacting the corresponding author (dmangale1@gmail.com).

## Appendix

**Table 6:** Annual total cost and unit cost per client-month of PrEP for the MOH scenario with inclusion of serum creatinine testing (2019 USD)

Costs (2019 USD)	MOH + Bi-annual Serum Creatinine Testing					
	Standard of care PrEP client visits (*N*=304); PrEP-months (*N*=913)			Six-month PrEP + HIVST PrEP client visits (*N*=340); PrEP-months (*N*=2039)		
Cost categories	Total annual cost	$ per client/month	% Total cost	Total annual cost	$ per client/month	% Total cost
Variable costs						
Personnel (clinical)	$4177.65	$4.57	31%	$3219.84	$1.58	13%
Medication	$6547.27	$7.17	49%	$14486.20	$7.11	59%
Laboratory testing	$875.36	$0.96	6%	4727.44	$2.32	19%
Recurrent training	$89.24	$0.10	1%	99.60	$0.05	0%
Supplies	$315.98	$0.35	2%	308.13	$0.15	1%
*Sub-total*	*$12005.50*	*$13.15*	*89%*	*22841.22*	*$11.20*	*94%*
Fixed costs						
Start-up-microplanning	$79.31	$0.09	1%	$88.53	$0.04	0%
Personnel	$277.32	$0.30	2%	$198.01	$0.10	1%
Capital	$375.06	$0.41	3%	$329.25	$0.16	1%
Overhead	$468.96	$0.51	3%	$523.45	$0.26	2%
Start-up training	$235.35	$0.26	2%	$372.24	$0.18	2%
*Sub-total*	*$1436.01*	*$1.57*	*10%*	*$1511.48*	*$0.74*	*6%*
Total costs	13479.33	$14.76	100%	$24352.70	$11.94	100%

## Abbreviations

HIV: Human Immunodeficiency Virus
PrEP: Pre-Exposure Prophylaxis
DSD: Differentiated Service Delivery
MOH: Kenyan Ministry of Health
HIVST: HIV Self-Test kit
OF: Oral Fluid
BB: Blood-based
USD: United States dollars
KSh: Kenya Shilling
PMTCT: Prevention of Mother to Child Transmission (PMTCT)
