# Supplementary material for: Opportunity for cost savings with a novel differentiated model of PrEP delivery: a comparative costing analysis of six-month PrEP supported by interim HIV self-testing and standard of care PrEP dispensing in Kenya
Source: BMC Health Serv Res. 2025 Jul 1;25:865. doi: 10.1186/s12913-025-12891-7 (PMC12220747; doi:10.1186/s12913-025-12891-7)
Supplement: Supplementary file 1 — Supplementary Material 1. [file 12913_2025_12891_MOESM1_ESM.docx]

Supplemental Information

**1. Cost categories informing the cost evaluation**

| **Recurrent** | *Personnel-Clinical* | Clinical personnel costs for PrEP initiation, HIV counselling and testing, and follow-up visits.  In the MOH scenario, public-sector salaries were replaced with salaries paid in the trial. |
| --- | --- | --- |
|  | *Drugs* | Cost of oral PrEP (co-formulated tenofovir disoproxil fumarate/emtricitabine) ($6.75 per 30 days) plus cost of storage and transportation costs (charged at 8% of the cost).  In the MOH scenario, the same drug costs were applied. |
|  | *Lab tests* | Included the cost of the OF HIV self-test kit, BB HIV self-test kit, rapid HIV test kit and buffer solution to confirm HIV-negative status for clients at initiation and follow-up visits as well as the cost of point-of-care creatinine test strips. Includes consumables required for test administration (e.g., gloves, lancets, etc.).  In the MOH scenario, we replaced the cost of point-of-care creatinine with the average amount for creatinine testing charged at public facilities. |
|  | *Other supplies* | Cost of appointment cards, client diary, prescription pads, PrEP encounter records, and other printing costs. In the MOH scenario, we applied the same supplies costs. |
| **Fixed** | *Start-up* | Fixed costs of microplanning, training, demand creating, sensitization and other activities. Examples are activities and resources associated with early-stage planning meetings to provide information and obtain consensus, generating interest for PrEP in the target population and among healthcare workers (demand creation and community mobilization).  In the MOH scenario, we assumed start-up costs were accumulated from high-level planning meetings with county and sub-county health officials in charge of HIV services (lab, counselling, testing and treatment, and pharmacy services) onboarding with facility leadership and training key staff. Also includes travel-related costs, materials and printing costs associated with the activities. |
|  | *Personnel: supervision & administration* | Staff costs spent on routine reporting, drug accounting, and meetings to debrief on PrEP delivery, service delivery management excluding research-specific activities.  In the MOH scenario, supervision and administration comprised the cost of quarterly refresher trainings and quarterly supervisory visits from county- and sub-county level health officers. |
|  | *Capital* | Cost of machines and equipment, including creatinine machines, control solutions, and furniture used during PrEP encounters. Years of useful life years was assumed to be five years for most items and one for items needing annual replacement.  In the MOH scenario, capital costs included the cost of machines and equipment, control solutions, and furniture. |
|  | *Overhead* | Costs included building, utilities, and maintenance costs multiplied by a fraction of PrEP visits out of total clinic visits. This also included transportation costs for facility visits for counsellor supervision, phones, airtime, printing costs for reporting tools.  In the MOH scenario, overhead costs included printing costs, building and utility costs as a fraction of PrEP visits expected out of total facility visits. We excluded any transportation and communication costs associated with routine supervision because this function is integrated into clinic flows and the nurse in-charge of the HIV clinic. |
