# Supplementary material for: Opportunity for cost savings with a novel differentiated model of PrEP delivery: a comparative costing analysis of six-month PrEP supported by interim HIV self-testing and standard of care PrEP dispensing in Kenya
Source: BMC Health Serv Res. 2025 Jul 1;25:865. doi: 10.1186/s12913-025-12891-7 (PMC12220747; doi:10.1186/s12913-025-12891-7)
Supplement: Supplementary file 2 — Supplementary Material 2. [file 12913_2025_12891_MOESM2_ESM.docx]

**JiPime JiPrEP Trial**

|  | In-depth Interview Guide for Costing |
| --- | --- |

Note: this list of questions will be adapted during observation period to encompass the locally-specific procedures, supplies, and materials used at each facility. Respondent may opt to follow-up with responses if unable to answer a question in the moment.

*Interviewer may skip over questions where answers are already known/ documented elsewhere.*

1. PERSONNEL
2. What is your job title and your role in the facility? (e.g., supervision, service delivery, system maintenance, etc.)
3. How many days do you work in a week and for how many hours do you work (i.e. when do you start work and when do you finish)?
4. Around what percentage of time do you spend on PREP DELIVERY activities in the last week?
5. Can you tell me what kinds of allowances you receive (for example housing, transport, etc)
6. Tell me about other staff that have been involved in the PREP DELIVERY activities. What are the positions/roles that cover those activities and how many staff are there in each position? What are their job titles and responsibilities?
7. DRUGS, TESTS AND LAB SUPPLIES
8. Please tell me all the drugs, lab tests for HIV/STIs and or office and lab supplies for viral load/CD4 and other supplies like rapid tests, STI tests, lancets and reagents used in PREP DELIVERY service. Provide details about the name, brand, quantity purchased each time or quantity per box/package purchased and dosage (adolescent/youth) available per package
   1. What are the unit costs of these items? If you are unable to tell us, who is the facility personnel who can provide this information?
      1. Who is responsible for paying the costs of these items? MOH or county government? JIPIME JIPREP project? Others? Please provide details about the others.
9. VEHICLE/TRANSPORT
10. Does the facility use any means of transport to provide or support the PREP DELIVERY service?
    1. If yes, tell me about the vehicle or whichever mode of transportation that is used (type, model, quantity, purchase value, year of purchase).
    2. Is it used for activities other than PREP DELIVERY service?
       1. If yes, what percentage of it is used for PREP DELIVERY service?
11. UTILITIES
12. What is the approximate monthly amount paid to cover the electric bill at this facility? Can you approximate what proportion of the electricity bill is due to activities related to PREP DELIVERY service? Ranges are okay.
13. What is the approximate monthly amount paid to cover the water bill at this facility? Can you approximate what proportion of the water bill is due to activities related to PREP DELIVERY service? Ranges are okay.
14. What is the approximate monthly amount paid to cover the phone bill at this facility? Can you approximate what proportion of the phone bill is due to activities related to PREP DELIVERY service? Ranges are okay.
    1. If applicable, can you tell us how credit or airtime for phone calls is disbursed to providers involved in PREP DELIVERY service? How much is provided and how frequently?
15. What is the approximate monthly amount paid to cover the internet bill at this facility? Can you approximate what proportion of the internet bill is due to activities related to PREP DELIVERY service? Ranges are okay.
16. What is the approximate monthly amount paid to cover the other utilities such as garbage collection and cleaning at this facility? Can you approximate what proportion of the garbage collection and cleaning is due to activities related to PREP DELIVERY service? Ranges are okay.
17. What is the approximate monthly amount paid to cover rent at this facility? Can you approximate what proportion of the rent is due to space occupied by PREP DELIVERY services? Ranges are okay. How many rooms are used for PREP DELIVERY? If rent rate is not available, what is the average rental cost for office space similar in size to the rooms used for PREP DELIVERY?
18. EQUIPMENT
19. Tell me about the equipment that are used for the intervention (type, model, quantity, purchase value, year of purchase). This includes things like fridges, large pieces of furniture, lab equipment etc.
20. Is it used for activities other than JIPIME JIPREP activities?
21. If yes, what percentage of it is used for JIPIME JIPREP?
